# 气管镜下介入治疗弥漫性气管混合性鳞状细胞和腺性乳头状瘤1例并文献复习

**DOI:** 10.3779/j.issn.1009-3419.2024.102.31

**Published:** 2024-09-20

**Authors:** Xiaosen HUO, Yao ZHANG, Yanyan DONG, Lei LI, Heng ZOU, Peng AN, Lingjie BIAN, Yuan LI, Hongwu WANG

**Affiliations:** ^1^101121 北京，北京中医药大学东直门医院呼吸病中心; ^1^Respiratory Disease Center, Dongzhimen Hospital of Beijing University of Chinese Medicine, Beijing 101121, China; ^2^101121 北京，北京中医药大学东直门医院病理科; ^2^Department of Pathology, Dongzhimen Hospital of Beijing University of Chinese Medicine, Beijing 101121, China

**Keywords:** 肺肿瘤, 混合性鳞状细胞和腺性乳头状瘤, 气管镜下介入治疗, Lung neoplasms, Mixed squamous cell and glandular papilloma, Bronchoscopic interventional treatment

## Abstract

肺混合性鳞状细胞和腺性乳头状瘤（mixed squamous cell and glandular papilloma, MSCGP）是肺乳头状瘤的一种亚型，依据生长部位分为中央型和外周型，以中央型较为多见，其临床表现主要与肿瘤生长部位有关。外科手术切除是肺MSCGP的主要治疗方式。气管内MSCGP接受气管镜下介入治疗能够获得满意的效果。现报道北京中医药大学东直门医院呼吸病中心收治的气管镜下介入治疗1例弥漫性气管MSCGP病例，以期了解其临床特征，为该类疾病的诊疗提供临床参考。

## Abstract (en)

Pulmonary mixed squamous cell and glandular papilloma (MSCGP) is a subtype of pulmonary papilloma, which can be classified as central type and peripheral type based on its site of development. The central type is the most common. The clinical manifestations of pulmonary MSCGP are mainly related to the location of the tumor. Surgery is the main treatment for this disease. Bronchoscopic interventional treatment for the MSCGP in the central trachea could receive satisfactory effect. We reported a patient suffered from diffuse tracheal MSCGP who was treated by bronchoscopic interventional treatment in Respiratory Disease Center, Dongzhimen Hospital of Beijing University of Chinese Medicine, aiming to enhance the recognition of the clinical features and provide clinical references for the diagnosis and treatment of such disease.

肺混合性鳞状细胞和腺性乳头状瘤（mixed squamous cell and glandular papilloma, MSCGP）是一种罕见的被覆鳞状上皮和腺上皮的良性肿瘤，是肺乳头状瘤的一种亚型，依据生长部位分为中央型和外周型，以中央型较为多见^[1,2]^。肺MSCGP的临床表现主要与肿瘤生长部位有关。当病变位于肺叶或段支气管近端时，患者可表现为胸闷、憋气及咳嗽等症状；而病变位于肺部周围时，患者可无任何症状。外科手术切除是治疗肺MSCGP的主要方式。发生在中央气管的MSCGP病例更为少见，仅见局限性生长的个案报道^[3]^。气管内MSCGP患者接受气管镜下介入治疗能够获得满意的效果。目前，弥漫性气管MSCGP病例未见文献报道。现报道北京中医药大学东直门医院呼吸病中心收治的气管镜下介入治疗弥漫性气管MSCGP病例1例，以期了解其临床特征，为该类疾病的诊疗提供临床参考。

## 1 病例资料

患者，男性，61岁，主因“间断咳嗽、咳黄色黏痰，伴喘憋13个月”入院。2022年1月初，患者间断出现咳嗽、咳黄色黏痰，无痰中带血丝，伴有喘憋，活动后加重，无发热。于2023年2月27日就诊于北京中医药大学东直门医院。吸烟史30余年，20支/d。体格检查：体温36.5 ^o^C，心率80次/min，呼吸20次/min，血压124/68 mmHg，口唇无紫绀，左肺呼吸音减弱，未闻及明显干湿啰音。卡氏体能状态（Karnofsky performance status, KPS）评分80分，气促评分2分。胸部增强计算机断层扫描（computed tomography, CT）示：气管隆突处见软组织影，气管下段及双侧主支气管管壁增厚，左主支气管狭窄，管径1-2 mm，狭窄面积80%-90%，狭窄长度约3 cm（图1A、1B），增强后明显强化。血气分析：pH 7.44，动脉血二氧化碳分压37.9 mmHg，动脉血氧分压77 mmHg，碱剩余1.4，血氧饱和度96.0%。肿瘤标志物：甲胎蛋白（α-fetoprotein, AFP）1.38 ng/mL，癌胚抗原（carcinoembryonic antigen, CEA）3.90 ng/mL，细胞角蛋白19片段（cytokeratin 19 fragment, CYFRA21-1）1.33 ng/mL，神经元特异性烯醇化酶（neuron specific enolase, NSE）10.34 ng/mL，鳞状上皮细胞癌相关抗原（squamous cell careinoma antigen, SCCA）0.80 ng/mL。全血细胞分析：白细胞10.38×10^9^/L，中性粒细胞百分比73.3%。初步诊断：中央型肺部肿瘤、中心气管狭窄III级、阻塞性肺炎和I型呼吸衰竭。本研究经北京中医药大学东直门医院伦理委员会审核批准（审批编号：2024DZMEC-286-01），患者签署知情同意书。

**图1:**
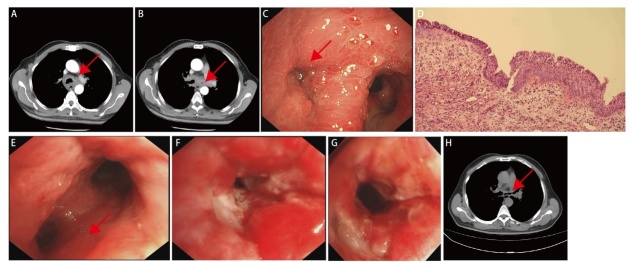
患者影像学资料、气管镜下治疗情况和肿瘤HE染色结果。A：气管下段管壁增厚（箭头）；B：双侧主支气管管壁增厚，左侧管腔严重狭窄（箭头）；C：VII和VIII区重度狭窄，病变弥漫性生长（箭头）；D：HE染色结果（×100）；E：治疗后左主支气管管腔轻度狭窄（箭头）；F：左主支气管管腔瘢痕性狭窄；G：激光消融和球囊扩张后左主支气管管腔轻度狭窄；H：气管壁水肿减轻和左主支气管轻度狭窄（箭头）。

积极控制肺部感染后，患者于2023年3月3日-29日进行多次气管镜介入治疗。全身麻醉后，高频喷射通气经硬质气管镜侧孔维持血氧饱和度。支气管镜经其后孔探查：中央气管下段（III区）见散在灰白色新生物；隆突部（IV区）和左主支气管（VII和VIII区）呈现弥漫性灰白色鱼鳞样新生物，向管腔内匍匐生长；VII和VIII区管径1-2 mm，属于重度狭窄（III级）（图1C）。活检钳分别钳取III、VII和VIII区病灶，采用氩气高频电刀烧灼和二氧化碳冷冻探头冻取结合的方法消除VII和VIII区病灶。氩气电刀接近病灶约3 mm时开始烧灼病灶，及时用活检钳钳出或二氧化碳冷冻探头冻取出碳化凝固的组织；二氧化碳冷冻探头贴近肿瘤表面，冷冻至其周围产生冰球，黏附肿瘤组织取出。治疗后，外径6 mm软质气管镜可通过左主支气管，充分灌洗并吸引支气管内脓性分泌物。术后病理（图1D）：III区黏膜急慢性炎症伴息肉样增生和局部病灶轻度不典型增生；VII和VIII区病灶呈现乳头及息肉状生长，表面被覆鳞状上皮和腺上皮，血管增生，伴有中等量炎性细胞浸润，符合MSCGP。免疫组织化学染色结果：P63（+）、P40（+）、细胞角蛋白5/6（cytokeratin 5/6, CK5/6）（+）、分化簇34（cluster designation 34, CD34）（脉管+）、CK7（部分+）、甲状腺转录因子1（thyroid transcription factor 1, TTF-1）（部分+）、新天冬氨酸蛋白酶A（Napsin A）（-）、CD163（散在+）、Ki-67（约5%+）、CD45（散在+）、抗细胞角蛋白1/3（anti-cytokeratin 1/3, AE1/3）（+）、波形蛋白（Vimentin）（+）、平滑肌激动蛋白（smooth muscle actin, SMA）（局部+）。鉴于气管管壁增厚和黏膜炎性反应，3月10日口服甲泼尼龙片对症治疗；初始剂量28 mg/d，并逐渐减量至8 mg/d。出院时，患者憋喘症状明显改善，左主支气管管腔恢复至I级狭窄，狭窄面积10%，管径约9 mm（图1E）。复查动脉血气，数值在正常范围。KPS评分70分，气促评分2分。5月5日，患者因胸闷及憋气再次入院。5月10日进行气管镜下探查示：VII区黏膜水肿及管腔II级狭窄，狭窄面积超过50%，管径约5 mm，远端管腔内大量黄白色分泌物淤积。气管镜灌洗并吸净管腔内分泌物，并于VII和VIII区水肿黏膜下注射2 mL复方倍他米松注射液，每处注射剂量以黏膜隆起为宜。术后患者症状缓解，甲泼尼龙片剂量由8 mg/d调整为4 mg/d，8周后停止口服。患者分别于2023年11月20日和2024年1月8日因喘憋加重两次入院。气管镜下探查示：VII和VIII区瘢痕形成，均属于管腔III级狭窄，狭窄面积分别为80%和70%，管径分别约2和3 mm（图1F）。多次进行激光消融膜部瘢痕和球囊扩张（10 mm×30 mm, 3 bar/min），并于黏膜下注射曲安奈德注射液40 mg，以黏膜隆起为宜。术后管腔恢复至I级狭窄，狭窄面积20%，管径约8 mm，气管壁水肿减轻（图1G、1H），症状缓解。2024年5月15日电话随访，患者未出现胸闷及憋气症状。

## 2 讨论

肺MSCGP是支气管乳头状瘤中占比最少的良性肿瘤类型，其病理组成同时具有鳞状上皮和腺上皮的乳头状瘤成分，且至少有1/3腺上皮覆盖^[4]^。以“肺混合性鳞状细胞和腺性乳头状瘤”“pulmonary mixed squamous cell and glandular papilloma”为检索词，在中国知网、万方和PubMed数据库中搜索2014至2024年文献27篇，共计51例。其中，13篇中文文献报道31例，14篇外文文献报道20例，所有患者均为病理确诊。剔除未明确肿瘤具体位置的3篇文献中的11例。结合本例和其余文献报道病例共计41例（失访1例和随访40例），随访时间2-52个月，未发现术后肿瘤转移及局部复发现象。具体情况见表1^[1⇓-3,5⇓⇓⇓⇓⇓⇓⇓⇓⇓⇓⇓⇓⇓⇓⇓⇓⇓⇓⇓-25]^，男女比例相当（男:女=21:20），以中老年为主，气管发病率最低，周围型居多。

**表1:** 已发表的肺混合性鳞状细胞和腺性乳头状瘤相关文献复习

Reference	Gender	Age (yr)	Smoking history	Lesion location	Tumor site	Boundary conditions of lesions
Saraya, et al.^[1]^	Female	17	No	Right inferior lobe	Central type	-
Li, et al.^[2]^	Female Female Male Male Male Male	57 67 49 48 39 59	No No No Yes No Yes	Right upper lobe Right upper lobe Right upper lobe Left inferior lobe Right upper lobe Right inferior lobe	Peripheral type Central type Peripheral type Peripheral type Peripheral type Peripheral type	Well-defined Well-defined Well-defined Well-defined Well-defined Well-defined
Luo, et al.^[3]^	Male	54	Yes	Trachea	Central type	-
Feng, et al.^[5]^	Female Male	65 60	No Yes	Right inferior lobe Right inferior lobe	Peripheral type Peripheral type	Ill-defined Ill-defined
Yabuki, et al.^[6]^	Male	76	Yes	Right inferior lobe	Peripheral type	-
Chen, et al.^[7]^	Female	68	No	Right inferior lobe	Peripheral type	-
Feng, et al.^[8]^	Male Female	65 63	Yes No	Left upper lobe Right upper lobe	Peripheral type Peripheral type	- -
Dong, et al.^[9]^	Female Female Male Male	44-80 (60)	No No Yes Yes	Right upper lobe Right upper lobe Right upper lobe Left inferior lobe	Central type Central type Peripheral type Peripheral type	Well-defined Well-defined Well-defined Well-defined
Abe, et al.^[10]^	Female	68	Yes	Left upper lobe	Peripheral type	-
Liu, et al.^[11]^	Female Male Male	48 11 70	No No Yes	Left inferior lobe Right middle lobe Left inferior lobe	Central type Central type Central type	Well-defined Well-defined Well-defined
Zheng, et al.^[12]^	Female Female Male Male	53-70	No No Yes No	Left upper lobe Left inferior lobe Right middle lobe Right inferior lobe	- - - -	Well-defined Well-defined Well-defined Well-defined
Chen, et al.^[13]^	Female	63	No	Right inferior lobe	Central type	-
Yang, et al.^[14]^	Female	53	-	Left inferior lobe	Central type	-
Wang, et al.^[15]^	Male	74	Yes	Right upper lobe	Peripheral type	-
Yun, et al.^[16]^	Female	64	No	Left inferior lobe	Peripheral type	-
Che, et al.^[17]^	Female Male	74 66	No -	Left inferior lobe Right upper lobe	Central type Central type	- Well-defined
Kawamoto, et al.^[18]^	Female	59	No	Left upper lobe	Central type	-
Iijima, et al.^[19]^	Male	49	Yes	Right inferior lobe	Peripheral type	-
Yang, et al.^[20]^	Female	57	No	Right inferior lobe	Peripheral type	Well-defined
Arora, et al.^[21]^	Female	77	No	Right inferior lobe	Peripheral type	-
Zhang, et al.^[22]^	Female	52	No	Right inferior lobe	Peripheral type	Well-defined
Yan, et al.^[23]^	Male	28	Yes	Left inferior lobe	Central type	-
Kawagishi, et al.^[24]^	Male	55	-	Right middle lobe	Peripheral type	-
Cao, et al.^[25]^	Male	67	Yes	Left upper lobe	Central type	-
This case	Male	61	Yes	Trachea	Central type	-
-: not reported.						

尽管有少数MSCGP病例在影像学或术后镜下观察时肿瘤与邻近正常组织之间边界不清晰^[5,6,26]^，但是组织学结构未出现极性消失和排列紊乱，同时肿瘤细胞也未出现异型性，例如核大、深染、核浆比例失调或病理性核分裂象等改变。这不仅解释了该肿瘤具备良性特征，而且也为其治疗方式提供了依据。外科手术切除是根治性治疗的主要方式，并且根据肿瘤的位置选择肺叶、肺段或楔形切除。本例患者的病变生长在临近隆突的两个区域，属于弥漫性中央气管腔内病变。外科需要进行支气管袖式切除术，应切除隆突和病变的左主支气管，然后进行隆突重建和气管端端吻合。该术式操作复杂，而且吻合口瘘、吻合口狭窄和肉芽肿形成等并发症是困扰胸外科医师的主要问题。瞿冀琛等^[27]^报道了胸腔镜下复杂袖式肺切除术后吻合口狭窄和吻合口瘘的发生率均为3.8%。气管镜下介入治疗具有创伤小、安全高效和并发症少的特点，已经广泛应用于气管内肿瘤治疗，尤其适合于良性病变。根据肿瘤的生长特性，可以选择以下方法^[28,29]^，如：氩气高频电刀切除、激光切除和二氧化碳冷冻消融等。患者首次入院后，针对“无蒂广基底”的病变特点，我们通过氩气高频电刀切除和二氧化碳冷冻消融的方法去除病灶，恢复了管腔通畅。

术后瘢痕性气道狭窄是良性气道狭窄的病因之一。气道黏膜损伤修复时分泌大量的细胞因子，参与炎症反应、肉芽组织增生及纤维化。另外，左主支气管管腔狭长，与气管夹角大，且易被主动脉弓挤压，也是形成瘢痕狭窄的不利因素。气管镜下介入治疗是治疗瘢痕性气道狭窄的有效方式^[30,31]^。方晓玉等^[32]^报道，气管镜介入治疗瘢痕性气道狭窄的有效率为80.9%。为减轻气管损伤和炎症反应，我们选择激光消融瘢痕。激光消融是一种精确定位、安全有效的消融方式，其热量传递对组织损伤范围小、深度浅，并使组织凝固、气化或碳化以达到消除病变的目的。松解瘢痕后，再辅以球囊扩张气道，均匀压力的扩张避免了膜部撕裂；同时适当延长扩张时间，以增加狭窄部承受的扩张压力，有利于缓解瘢痕狭窄。糖皮质激素具有抗炎和抗纤维化的作用^[33]^，口服和局部注射可抑制瘢痕再狭窄。治疗后患者气道管壁水肿明显减轻。

随访过程中，本例患者治疗后出现了3次不同时间点的再狭窄现象，且狭窄程度不同，这为术后随访时间指导带来了困难。大部分瘢痕性气道狭窄患者需要接受多次气管镜介入治疗^[34]^，因此，对于该特殊病例，我们建议随访应以症状为主导进行管理，提高治疗效果。

综上，对于弥漫性气管病变的MSCGP，考虑到病变无明显恶性潜能和外科手术创伤风险的情况，选择气管镜下介入治疗是一次突破性尝试。尽管瘢痕性气道狭窄是良性气道狭窄的并发症，但是气管镜介入治疗的合理应用和按需随访可提高其治疗效果。
